# 2,3‐Dichloro‐5,6‐Dicyano‐1,4‐Benzoquinone (DDQ)‐Mediated C—C Bond Formation: Redox Strategies from Stoichiometric to Catalytic Systems

**DOI:** 10.1002/open.202500568

**Published:** 2025-11-27

**Authors:** Dohoon Cha, Sun‐Joon Min

**Affiliations:** ^1^ Department of Applied Chemistry Hanyang University (ERICA) Ansan Republic of Korea; ^2^ Center for Bionano Intelligence Education and Research Hanyang University (ERICA) Ansan Republic of Korea; ^3^ Department of Energy and Bio Sciences Hanyang University (ERICA) Ansan Republic of Korea

**Keywords:** 2,3‐dichloro‐5,6‐dicyano‐1,4‐benzoquinone, C—C bond formation, catalytic, stoichiometric

## Abstract

2,3‐Dichloro‐5,6‐dicyano‐1,4‐benzoquinone (DDQ) has long been recognized as a versatile organic oxidant that mediates diverse transformations through single‐electron transfer, hydride abstraction, and redox cycling. Beyond its classical stoichiometric role in oxidation and dehydrogenation, DDQ now serves as an efficient catalyst for carbon–carbon bond formation across thermal, photochemical, and electrochemical domains. In stoichiometric reactions, DDQ enables benzylic and allylic C—H activation to generate oxocarbenium or iminium intermediates that couple with a broad range of nucleophiles, facilitating alkylation, arylation, cyanation, and annulation processes. In catalytic systems, DDQ participates in redox cycles where the DDQ/DDQH_2_ couple is regenerated by oxidants such as O_2_, nitrites, or MnO_2_, offering mild and simple access to complex carbon frameworks. The scope further extends to asymmetric catalysis and radical‐mediated cross‐dehydrogenative coupling, providing sustainable routes to natural product‐like scaffolds and biologically active molecules. This review highlights the progression of DDQ from a stoichiometric oxidant to a redox‐active catalyst, emphasizing its growing utility in controlled, metal‐free oxidative C—C bond formation and its promise for next‐generation sustainable synthesis.

## Graphical abstract

2,3‐Dichloro‐5,6‐dicyano‐1,4‐benzoquinone serves as a versatile oxidant and redox catalyst for C—C bond formation through hydride abstraction or single‐electron transfer. Stoichiometric and catalytic systems enable benzylic, allylic, and aromatic C—H activation under mild, metal‐free conditions, providing sustainable routes to complex and bioactive molecules.

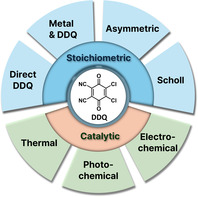

## 1 Introduction

**SCHEME 1:**
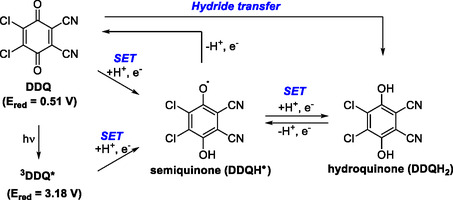
The structure of DDQ and its oxidation potential.

2,3‐Dichloro‐5,6‐dicyano‐1,4‐benzoquinone (DDQ) is a potent and versatile oxidant widely employed in organic synthesis [1]. It has proven effective in diverse transformations, including the oxidation of alcohols [2], dehydrogenative aromatization [3], and the construction of carbon–carbon [4], carbon–oxygen, and carbon–nitrogen bonds [1, 5, 6]. Many of these reactions proceed under mild conditions, making DDQ compatible with sensitive functional groups. The synthetic utility of DDQ is fundamentally derived from its unique electronic structure, which permits three accessible redox states: the oxidized quinone, the one‐electron‐reduced semiquinone, and the two‐electron‐reduced hydroquinone. This redox flexibility enables DDQ to engage in both ionic and radical reaction pathways [1, 2]. In its ground state, DDQ exhibits an oxidation potential of 0.51 V versus SCE. Notably, its one‐electron oxidizing capacity is significantly enhanced under visible‐light excitation, reaching 3.18 V versus SCE in the triplet excited state (^3^DDQ) and 3.8 V versus SCE in the singlet excited state (^1^DDQ) [7]. Owing to these unusual redox properties, DDQ has played a pivotal role in the advancement of novel synthetic methodologies and in the synthesis of complex molecular scaffolds (Scheme 1) [8].

Mechanistically, DDQ frequently promotes reactions through hydride transfer, abstracting a hydride ion from activated C—H bonds and thereby generating carbocation intermediates [1, 2]. These intermediates can undergo a wide range of subsequent transformations, including intermolecular couplings [9] and intramolecular cyclizations [10], which are highly valued in the synthesis of organic materials and chemical libraries. More recently, photochemical studies have demonstrated that, in its excited state, DDQ readily engages in single‐electron transfer (SET) processes, oxidizing substrates to radical cations that subsequently undergo productive transformations [7]. In certain cases, the reduced form of DDQ can be reoxidized to regenerate the oxidant, thereby enabling catalytic turnover. This mechanistic versatility has facilitated the development of highly efficient strategies for constructing synthetically valuable compounds that are often inaccessible with alternative oxidants (Scheme 1).

DDQ‐mediated C—C bond formation can be broadly categorized into stoichiometric and catalytic approaches. In stoichiometric methods, DDQ is most commonly applied in oxidative C—H activation [1]. Cross‐dehydrogenative coupling (CDC) reactions, pioneered by Li and coworkers, illustrate that benzylic, allylic, and aryl C—H bonds can be efficiently activated to undergo C—C bond formation [11]. Alternatively, DDQ serves as a stoichiometric co‐oxidant in the presence of metal catalysts, either facilitating Lewis acid catalysis or promoting electron transfer to induce bond construction [9]. Radical‐based C—C bond‐forming processes, exemplified by the Scholl reaction, involve DDQ‐mediated generation of radical‐cation species from aromatic substrates, which subsequently couple to produce extended aromatic frameworks [8].

In contrast to these stoichiometric strategies, catalytic applications of DDQ have further broadened its synthetic utility, enabling the development of more convenient and sustainable chemical processes. DDQ can act as an organocatalyst in aerobic oxidations, often in combination with mediators such as nitrite or metal oxides (MnO_2_, PbO_2_) [2, 7]. As a photocatalyst, DDQ absorbs visible light and activates substrates through excited‐state electron transfer, thereby promoting C—C bond formation under milder conditions with improved selectivity for complex molecules. In electrochemical applications, DDQ catalysis has been more extensively established for C—N and C—O bond construction, whereas examples of C—C bond formation remain relatively unexplored, though mechanistic and methodological studies are gradually being pursued [6]. The principal challenge with achieving C—C bond formation under electrochemical DDQ catalysis lies in stabilizing and controlling reactive intermediates, which continues to be an active area of investigation.

This review aims to provide a comprehensive survey of DDQ‐mediated and catalyzed C—C bond formation reactions. It will be focused on the scope and limitations of these transformations, with discussion of proposed reaction mechanisms and strategic innovations permitting selective carbon–carbon bond coupling. Advances in mechanistic understanding, substrate scope, and synthetic utility highlight the importance and potential of DDQ to broaden strategies for molecular construction in organic synthesis.

## 2 DDQ‐Mediated C—C Bond Formation

### 2.1 Direct DDQ‐Mediated C—C Bond Formation

A stoichiometric amount of DDQ can be used to directly oxidize the α‐C—H bond of either oxygen or nitrogen in ethers or amines to generate cationic intermediate, which subsequently react with various carbon nucleophiles to afford α‐substituted ether or amine derivatives.

**SCHEME 2:**
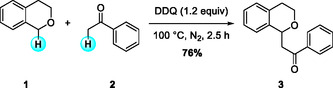
DDQ‐mediated, metal‐free CDC of isochroman with simple ketones.

In 2006, Zhang and Li first reported a DDQ‐mediated, metal‐free CDC reaction of benzyl ethers with simple ketones. They demonstrated isochromans and benzyl methyl ether were reacted with a broad range of substrates including aliphatic and aromatic ketones to afford a reasonable yield of products (Scheme 2) [11]. Isochroman showed relatively higher reactivity than simple benzyl methyl ether. The protocol was simplified, requiring only DDQ under solvent‐free (neat) conditions, as preliminary solvent screenings had shown variable performance.

**SCHEME 3:**
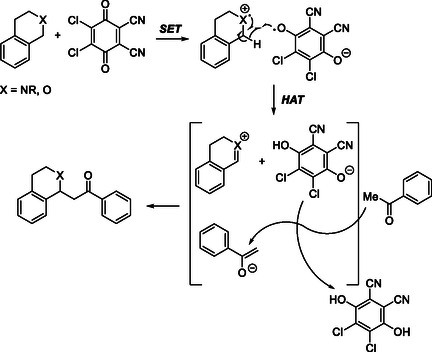
Proposed mechanism for DDQ‐mediated, metal‐free CDC reaction of isochroman with ketone.

Mechanistically, a SET from the oxygen of benzyl ether to DDQ generates a radical cation and DDQ radical anion. Then, a hydrogen atom transfer (HAT) from radical cation to DDQ radical anion generates an oxonium cation. The DDQ anionic oxygen abstracts α‐position proton to generates enolate. Finally, enolate attack the benzoxy cation to generates the CDC product by forming a new C—C bond (Scheme 3).

**SCHEME 4:**
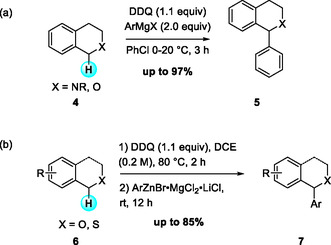
Sp^3^ C—H bond arylation of THIQ and isochromans with organometallic reagents. (a) Arylation with Grignard reagents; (b) arylation with arylzinc reagents.

Since the first DDQ‐mediated CDC reaction was reported, various types of carbon nucleophiles have been investigated for oxidative C—C bond‐forming reactions. In 2013, Muramatsu and coworkers described the arylation of sp^3^ C—H bonds in tetrahydroisoquinoline (THIQ) and isochroman via nucleophilic addition of aryl Grignard reagents to iminium or oxonium ions generated through DDQ oxidation (Scheme 4a) [12]. The method highlighted heavy‐metal‐free, highly regioselective benzylic arylation of THIQs and isochromans under mild conditions within a short reaction time. The reaction tolerated diverse N‐protected THIQ and isochroman derivatives as well as substituted aryl Grignard reagents. Mechanistic studies suggested that hydrogen abstraction from the radical‐cation intermediate is the rate‐determining step, as previously supported by the kinetic isotope effect [13].

In 2018, Peng et al. further expanded this methodology by developing a DDQ‐mediated oxidative sp^3^ C—H arylation of benzyl ethers with Knochel‐type arylzinc reagents (Scheme 4b) [14]. Their study provided concise access to 1‐aryl benzyl ethers through a DDQ‐initiated, C(sp^3^)—H arylation, in which DDQ serves to generate benzylic oxonium intermediates under mild conditions with functional‐group tolerance.

**SCHEME 5:**
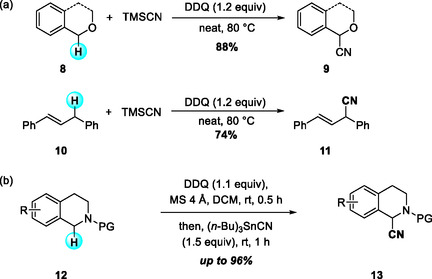
Sp^3^ C—H bond cyanation of benzyl ethers, 1,3‐diarylpropenes, and N‐protected THIQs. (a) DDQ‐mediated cyanation of benzyle ether and 1,3‐diarylpropene; (b) DDQ‐mediated cyanation of N‐protected THIQs.

In 2015, Wang and coworkers reported a DDQ‐mediated direct cyanation of benzyl ethers and 1,3‐diarylpropenes with trimethylsilyl cyanide (TMSCN) via functionalization of sp^3^ C—H bonds adjacent to heteroatoms or allylic benzylic carbons (Scheme 5a). This study established a general, DDQ‐promoted, metal‐ and solvent‐free cyanative coupling of benzylic sp^3^ C—H donors, providing an efficient approach to nitrile synthesis under mild conditions [15]. The methodology was further extended to benzyl methyl ether and dihydroisobenzofuran, enabling α‐functionalization reactions with broad synthetic applicability. Similarly, Lee et al. report the development of a complementary cyanation protocol employing tributylstannyl cyanide (Bu_3_SnCN) as the CN source for DDQ‐mediated α‐cyanation of various N‐acyl and N‐sulfonyl THIQs (Scheme 5b) [16]. The use of Bu_3_SnCN allowed for efficient transfer of the cyano group to nitrogen‐protected heterocycles, overcoming the limited reactivity of TMSCN in such systems. This metal‐free approach exhibited broad substrate tolerance and provided a versatile platform for streamlined access to bioactive heterocyclic motifs.

**SCHEME 6:**
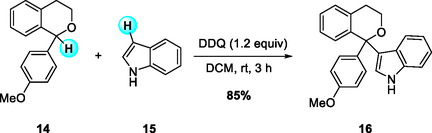
Formation of α‐aryl isochromanes with quaternary centers via CDC reaction.

Stoichiometric DDQ oxidation has also been exploited for the construction of quaternary carbon centers through carbon–carbon bond formation. In 2019, Ma and Liu et al. reported a CDC reaction that forms C(sp^2^)—C(sp^3^) bonds under mild conditions by directly coupling secondary benzyl ethers‐particularly α‐aryl isochromanes with indoles and pyrroles (Scheme 6) [17]. This synthetic methodology leads to the rapid generation of a diverse isochromane library featuring various α‐aryl‐heteroaryl substitution patterns, which may serve as useful intermediates for the discovery of biologically active compounds. Moreover, the successful demonstration of the reaction on a gram scale highlights its practicality and potential for synthetic applications.

**SCHEME 7:**
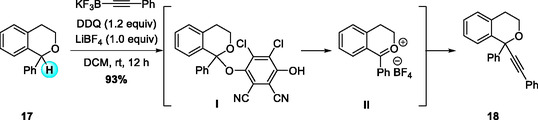
DDQ‐mediated sp^3^ C—H alkynylation of α‐substituted isochromans.

Following their work on CDC reactions, the same research group reported the first practical bimolecular oxidative C—H alkynylation of α‐substituted isochromans using potassium alkynyltrifluoroborates (Scheme 7) [18]. This method efficiently produced tertiary ethers bearing α‐quaternary stereocenters under mild, metal‐free conditions. The reaction proceeds through DDQ oxidation to generate a phenolate‐type ketal intermediate **I**, which then collapses in the presence of LiBF_4_ to form a 1,1‐disubstituted oxocarbenium ion **II** that is trapped by the alkynyl‐BF_3_K nucleophile. Systematic additive screening revealed LiBF_4_ as the optimal promoter for this transformation. The method showed a broad substrate scope, accommodating α‐aryl, heteroaryl, and alkyl isochromans as well as diverse aryl, heteroaryl, and alkyl alkynes. The reaction could also be performed on a gram scale without loss of efficiency, and the incorporated alkyne moiety serves as a versatile functional handle for further derivatization.

**SCHEME 8:**
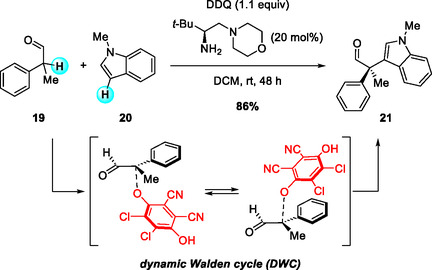
Aminocatalytic DDQ‐mediated oxidative coupling of α‐branched aldehydes.

Subsequently, Jørgensen, Houk, and coworkers in 2021 reported an asymmetric S*_N_* 2‐type dynamic kinetic resolution (DKR) reaction that forms quaternary stereocenters from α‐branched aldehydes (Scheme 8) [19]. In this transformation, an O‐bound quinol group, introduced through a DDQ‐mediated two‐electron oxidation, serves as a leaving group in an enantioselective S_*N*_2 substitution with indoles catalyzed by a chiral amine. Computational and kinetic studies confirmed that the chiral amine catalyst promotes both racemization and enantioselective substitution through a dynamic Walden cycle, enabling DDQ‐activated intermediates to undergo S_*N*_2 reactions that generate quaternary stereocenters with high enantioselectivity. Consequently, DDQ plays a dual role as both oxidant and leaving‐group precursor, allowing the efficient formation of highly enantioenriched quaternary centers that would otherwise be difficult to access without a metal catalyst.

**SCHEME 9:**
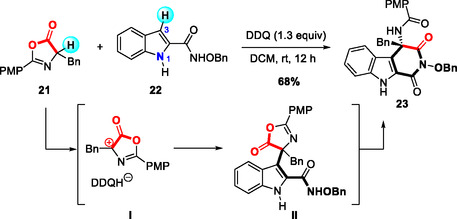
Umpolung [4 + 2] cyclization of azlactone and indole‐2‐amide.

In 2024, Yang, Huang, and coworkers reported a DDQ‐mediated umpolung [4 + 2] cyclization between azlactones and indole‐2‐amides (Scheme 9) [20]. Reaction optimization revealed that C3‐selective products were predominantly formed in the presence of DDQ at room temperature, with no N‐selective product observed. Other oxidants failed to promote this transformation, and the addition of metal salts was found to inhibit the reaction. The proposed mechanism involves initial DDQ‐induced homocoupling (not shown), followed by heterolytic cleavage to generate a highly reactive cationic species I. Subsequent nucleophilic attack and intramolecular aminolysis of this intermediate afford the corresponding cyclized products.

**SCHEME 10:**
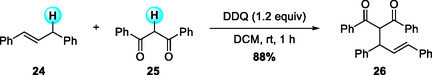
DDQ‐mediated CDC reaction of diarylallylic sp^3^ C—H bonds with 1,3‐dicarbonyl derivatives.

In contrast to heteroatom‐stabilized substrates, systems bearing benzylic or allylic C—H bonds without adjacent heteroatoms (unlike benzyl ether or amines) have also been investigated for DDQ‐promoted C—C bond‐forming reactions. In 2008, Bao and Cheng reported a DDQ‐mediated CDC reaction of diarylallylic sp^3^ C—H bonds with the active methylenic sp^3^ C—H bonds of 1,3‐dicarbonyl derivatives (Scheme 10) [21]. The solvent system played a crucial role in controlling both reaction rate and efficiency. The best yield was obtained when the reaction was conducted with 1.2 equivalents each of 1,3‐diphenylpropene and DDQ in dichloromethane at ambient temperature for 1 h. This metal‐free oxidative coupling provides an attractive alternative to palladium‐catalyzed allylic alkylation, highlighting the potential of DDQ as a mild and efficient oxidant for allylic activation.

**SCHEME 11:**
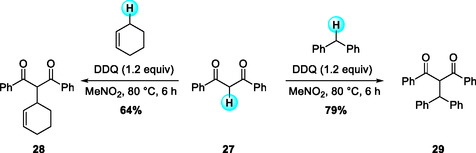
DDQ‐mediated oxidative C—C coupling of benzylic and allylic sp^3^ C—H with 1,3‐dicarbonyls.

Following this study, in 2010, Venkateswarlu and coworkers reported a related DDQ‐mediated oxidative C—C coupling between benzylic sp^3^ C—H donors and the active methylene groups of 1,3‐dicarbonyl compounds (Scheme 11) [22]. In this case, the reaction was performed in nitromethane at 80°C for 6 h, demonstrating that elevated temperature and a more polar solvent enhanced the coupling efficiency for less reactive benzylic substrates. The reaction proceeds through DDQ‐induced dehydrogenative activation, which constitutes the key step of this transformation, enabling operational simplicity and atom‐economical C—C bond formation. Interestingly, less activated substrates such as cyclohexene, cyclopentene, and tetrahydronaphthalenes were also compatible under the optimized conditions, effectively affording the desired products.

**SCHEME 12:**
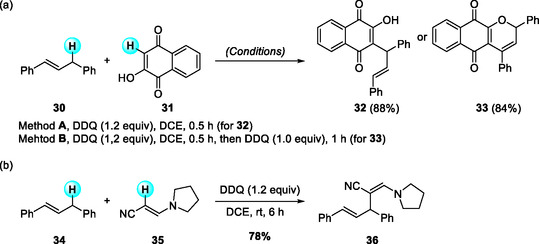
DDQ‐mediated CDC reactions of 2‐hydroxy‐1,4‐naphthoquinone (lawsone) (a) and push–pull enamines (b) with 1,3‐diarylpropenes.

In 2017, Cheng and coworkers further developed a metal‐free CDC and subsequent cyclization between the C(sp^2^)—H bond of 1,4‐naphthoquinone (lawsone) and the allylic C(sp^3^)—H bond of 1,3‐diarylpropenes (Scheme 12a) [23]. The reaction proceeded efficiently under mild, catalyst‐free conditions in dichloroethane, affording 3‐substituted 1,4‐naphthoquinones and pyranonaphthoquinones in good yields. Mechanistic studies, including TEMPO inhibition experiments, confirmed a SET pathway in which DDQ generates an allylic cation intermediate that couples with the C‐3 position of lawsone to furnish the coupled products, which subsequently undergo DDQ‐promoted intramolecular cyclization to form pyranonaphthoquinone derivatives.

Later that year, Cheng's group also disclosed a DDQ‐mediated α‐alkylation of enamines with 1,3‐diarylpropenes (Scheme 12b) [24]. The reaction exhibited broad substrate tolerance, accommodating various push–pull enamines, while asymmetric diarylpropenes produced the expected α/γ isomeric mixtures consistent with allylic cation trapping. α‐Oxo ketene dithioketal derivatives were likewise employed as alternative push–pull alkenes, further expanding the reaction scope. Collectively, these studies represent typical examples of DDQ‐mediated coupling between C(sp^3^)—H and C(sp^2^)—H bonds, providing an atom‐economical route to functionalized allylated products.

**SCHEME 13:**
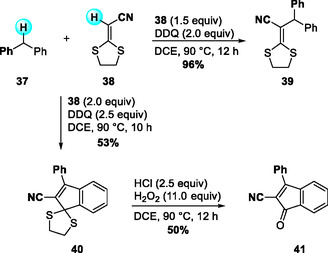
DDQ‐mediated intermolecular CDC reaction of benzylic sp^3^ C—H with vinylic sp^2^ C—H bonds.

Earlier, in 2014, Zhao and Liu had introduced dithioketals as effective substrates in DDQ‐mediated CDC reactions. In that study, they reported a DDQ‐mediated intermolecular CDC reaction between benzylic sp^3^ C—H donors and the vinylic sp^2^ C—H bonds of alkenes (Scheme 13) [25]. By varying the amounts of alkene and DDQ, the reaction selectively afforded either CDC products or indene thioketals.

**SCHEME 14:**
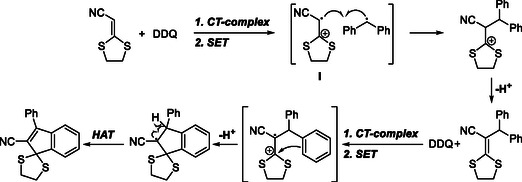
Proposed mechanism for CDC reaction of benzylic sp^3^ C—H with vinylic sp^2^ C—H bonds.

The proposed mechanism involves the formation of a charge‐transfer complex followed by a SET to generate a radical ion pair **I**. The resulting intermediate **I** then reacts with a benzylic radical to form a cationic species, which undergoes elimination to produce the coupled alkene product. In the presence of excess DDQ, further oxidation and intramolecular cyclization occur to yield the corresponding indenes. Additionally, the obtained indene dithioketals were readily converted to indanones via hydrolysis (Scheme 14).

**SCHEME 15:**
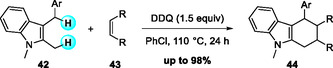
Dehydrogenative Diels–Alder (DDA) of 2‐methyl‐3‐arylmethylindoles.

The broad synthetic utility of DDQ in oxidative C—C bond formation has also been demonstrated in cycloaddition reactions. In 2015, Zhou, Xu, and Zhang reported a DDQ‐mediated dehydrogenative Diels–Alder (DDA) reaction of 2‐methyl‐3‐arylmethylindoles with electron‐deficient dienophiles (Scheme 15) [26]. Screening of oxidants and solvents revealed DDQ as the most effective oxidant and chlorobenzene as the optimal solvent. The protocol features benzylic C—H activation, ring formation and easy procedure, providing rapid access to carbazole and heteroacenes scaffolds from simple indole precursors.

**SCHEME 16:**
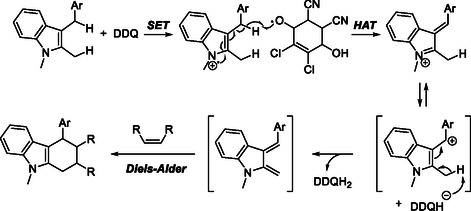
Proposed mechanism for DDA of 2‐methyl‐3‐arylmethylindoles.

Mechanistic studies showed that DDQ serves as the terminal oxidant, initiating benzyl sp^3^ C—H activation of 2‐methyl‐3‐arylmethylindole via sequential SET and HAT processes. These steps generate a benzyl cation intermediate along with the reduced quinone species (DDQH/DDQH_2_). Subsequent deprotonation by the reduced quinone produces a stereospecific indole‐derived *ortho*‐quinodimethane (*o*QDM) intermediate, which undergoes a [4 + 2] cycloaddition with electron‐deficient dienophiles to afford the tetrahydrocarbazole skeleton (Scheme 16). This cascade efficiently constructs two C—C bonds through direct sp^3^ C—H functionalization under mild conditions.

**SCHEME 17:**
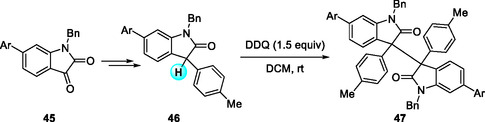
DDQ‐mediated oxidative dimerization of oxindolyl precursors.

In 2024, Chi and coworkers reported a DDQ‐mediated oxidative dimerization that converts oxindolyl precursors into σ‐dimerized oxindolyl dimers under mild conditions, yielding materials with tunable thermo‐ and mechanochromic properties (Scheme 17) [27]. Radicals are generated from the reduced precursors at room temperature through DDQ oxidation, and the resulting monomers readily dimerize, as confirmed by low‐temperature NMR and X‐ray analysis. When heated in polar solvents, the equilibrium shifts toward the monomer, producing a reversible color change from colorless to deep green. Mechanical grinding in the solid state similarly enhances ESR intensity and green coloration, consistent with force‐induced σ‐bond cleavage. This work establishes a versatile DDQ‐based radical platform exhibiting reversible color switching and sensitivity to thermal, mechanical, and solvent stimuli. The straightforward oxidative configuration and reversible radical dimerization offer a general strategy for designing functional materials.

**SCHEME 18:**
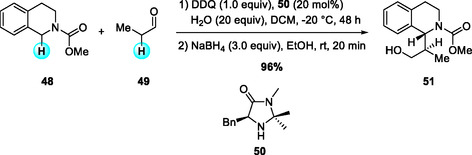
Organocatalytic enantioselective CDC of N‐carbamoyl cyclic amines with aldehydes.

In 2016, Liu and coworkers reported an organocatalytic enantioselective CDC of N‐carbamoyl cyclic amines with aldehydes using DDQ as the key oxidant (Scheme 18) [28]. N‐carbamoyl THIQs are first oxidized by DDQ and then coupled with aldehydes in the presence of optically active imidazolidinone salts (A·TFA) (**50**), affording enantiomerically enriched adducts bearing two adjacent stereogenic centers with excellent enantioselectivity but only moderate diastereoselectivity. The reaction sequence involves initial oxidation of the amine at ambient temperature, followed by enantioselective induction mediated by the chiral catalyst and aldehyde at low temperature to promote effective asymmetric control. The resulting products can be further derivatized through oxazolidine formation and subsequent hydrolysis to amino alcohols, or reduction to N‐methyl THIQ, without loss of optical purity, highlighting the synthetic utility of this transformation.

**SCHEME 19:**
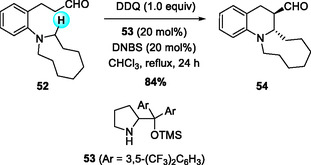
Organocatalytic synthesis of THQs via DDQ oxidation/1,5‐hydride transfer (1,5‐HT)/cyclization sequences.

Complementary to intermolecular reaction, intramolecular cyclization reactions via DDQ‐mediated C—C bond formation have proven effective for building stereochemically defined heterocycles. In 2013, Kim and coworkers reported the synthesis of fused tetrahydroquinolines (THQs) through a DDQ‐mediated oxidative enamine 1,5‐hydride transfer (1,5‐HT)/cyclization cascade (Scheme 19) [29]. The combination of diarylprolinol silyl ether (**53**) and 2,4‐dinitrobenzenesulfonic acid (DNBS) delivered moderate to high yields and good diastereoselectivity across five‐ to nine‐membered azacyclic rings, whereas bicyclic variants exhibited lower diastereomeric ratios (dr ≈ 1.2:1).

**SCHEME 20:**
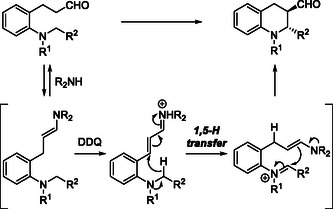
Plausible mechanism for this process.

Mechanistically, the key feature of this cascade lies in the generation of a cationic intermediate by DDQ oxidation, which triggers the 1,5‐HT and subsequent ring closure. The 3‐(*o*‐dialkylaminoaryl)propanals are first converted to enamines by the chiral organocatalyst, and the resulting enamines are oxidized by DDQ to produce α,β‐unsaturated iminium cation species. The ensuing 1,5‐HT/cyclization sequence furnishes the fused THQ framework with high stereoselectivity (Scheme 20).

**SCHEME 21:**
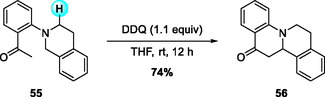
DDQ‐mediated intramolecular oxidative alkylation of N‐aryl THIQs.

In addition, Wang and coworkers in 2013 described a DDQ‐mediated intramolecular oxidative alkylation for the synthesis of ring‐fused THQs (Scheme 21) [30]. After screening a range of organic and inorganic oxidants, they found that the reaction proceeded most efficiently using a stoichiometric amount of DDQ in THF at room temperature. In this system, N‐aryl THIQs served as the primary substrates, and electron‐rich THIQ derivatives generally afforded higher yields. This protocol provides a straightforward and transition‐metal‐free route for the intramolecular construction of fused THQ frameworks via single‐step C(sp^3^)—C(sp^3^) bond coupling.

**SCHEME 22:**
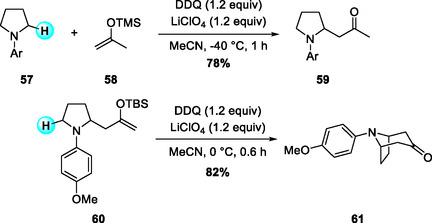
Sequential DDQ‐mediated oxidative Mannich coupling reactions for synthesis of 8‐azabicyclo[3.2.1]octanes.

In 2018, Min and coworkers reported the synthesis of 8‐azabicyclo[3.2.1]octanes through a sequential DDQ‐mediated oxidative Mannich coupling sequence comprising an initial intermolecular coupling between N‐aryl pyrrolidines and a TMS enol ether, followed by intramolecular oxidative Mannich cyclization of the resulting silyl enol ether (Scheme 22) [31]. The reaction proceeds smoothly under mild conditions, exhibiting broad functional‐group tolerance toward both electron‐rich and amphiphilic aryl substituents. Notably, this strategy provided an efficient and concise synthesis of atropine‐type alkaloid derivatives, highlighting its potential as a practical route to structurally complex tropane frameworks.

DDQ serves as a key oxidant for converting sp^3^ C—H bonds‐particularly in benzyl, allylic, and related substrates‐into reactive cationic intermediates such as oxocarbenium or iminium ions, thereby promoting direct coupling with diverse carbon nucleophiles. The resulting CDC reactions exemplify a metal‐ and ligand‐free design, often employing a single oxidant, as demonstrated in numerous studies from the 2010s. This approach features excellent step and atom economy as well as notable solvent and oxidant selectivity. The incorporation of carbon nucleophiles such as Grignard reagents, aryl and alkynyl organometallics, and cyanides has greatly expanded its synthetic versatility. Subsequent developments extended this strategy to include S_*N*_2‐type DKR (quinolyl leaving group installation followed by S_*N*_2 displacement via two‐electron oxidation) and DDQ‐induced umpolung [4 + 2] cyclization processes. Collectively, these advances establish a modular C—C bond‐forming platform that enables the efficient construction of complex molecular skeletons and tertiary or quaternary carbon centers under mild conditions.

### 2.2 Catalytic DDQ‐Assisted C—C Bond Formation

In many oxidative coupling reactions, transition‐metal catalysts such as copper and iron serve as the primary oxidants for C—H activation, while DDQ is employed as a stoichiometric co‐oxidant to maintain the catalytic cycle. In other systems, DDQ in combination with a Brønsted acid can facilitate single‐electron oxidation of the substrate to generate radical‐cation intermediates, which subsequently participate in C—C bond‐forming processes.

**SCHEME 23:**
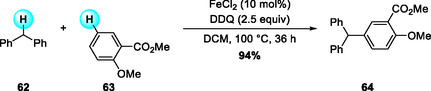
FeCl_2_‐catalyzed cross‐dehydrogenative arylation (CDA) of benzylic sp^3^ C—H with electron‐rich arenes.

In 2009, Shi and coworkers reported an FeCl_2_‐catalyzed, DDQ‐mediated cross‐dehydrogenative arylation (CDA) that couples benzylic sp^3^ C—H donors with electron‐rich arenes to afford triarylmethanes with high chemo‐ and regioselectivity (Scheme 23) [32]. Among various oxidants and metals examined, DDQ proved superior to peroxides, and iron outperformed other transition metals.

**SCHEME 24:**
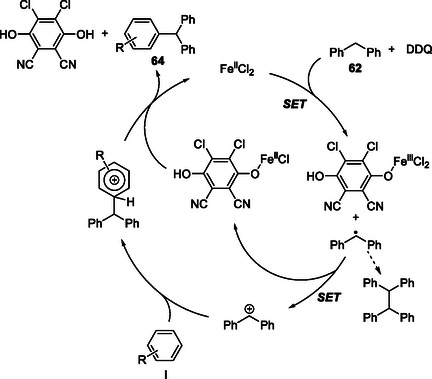
Proposed mechanism for FeCl_2_‐catalyzed CDA.

The mechanistic studies revealed a cooperative redox interplay between DDQ and iron in activating the benzylic C—H bond. The FeCl_2_/DDQ system promotes a two‐step SET oxidation of the benzylic donor **62**: the first SET generates a benzylic radical, followed by a second oxidation to form the corresponding benzyl cation. This cation undergoes electrophilic aromatic substitution with **I** to form a Wheland *σ*‐complex, which rearomatizes by deprotonation with DDQH^−^ as the base to yield the triarylmethane coupling product **64**. During this catalytic cycle, iron mediates electron transfer between DDQ and its reduced form, thereby regenerating DDQ and completing the DDQ/Fe redox process. This Fe/DDQ arylation reaction demonstrated that diphenylmethane derivatives are effective benzyl sp^3^ C—H donors under oxidative conditions (Scheme 24).

**SCHEME 25:**
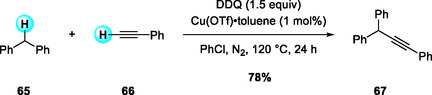
DDQ‐promoted, CuOTf‐catalyzed CDC reactions between terminal aryl alkynes and diphenylmethane derivatives.

Subsequently, in 2010, Correia and Li reported a DDQ‐promoted, CuOTf‐catalyzed CDC between terminal aryl alkynes and the benzylic sp^3^ C—H bonds of diphenylmethane derivatives, affording disubstituted alkynes (Scheme 25) [33]. The substrate scope revealed that aliphatic alkynes were unreactive, electron‐rich alkynes gave diminished yields, and 3‐fluorophenylacetylene exhibited low reactivity due to reduced nucleophilicity. This study represented the first example of a metal‐catalyzed alkynylation of benzylic C—H bonds not α to nitrogen. The CuOTf/DDQ system effectively expanded the reactivity profile of diphenylmethane donors from arylation to alkynylation, further providing complementary couplings with heteroarenes.

**SCHEME 26:**
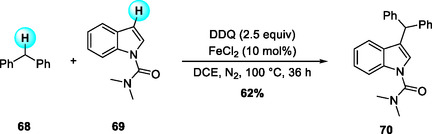
FeCl_2_‐catalyzed CDC of diphenylmethane derivatives with indoles.

Thereafter, in 2015, Chen and colleagues developed a FeCl_2_‐catalyzed CDC that achieves C3‐alkylation of indoles using diphenylmethane derivatives as benzylic donors (Scheme 26) [34]. Mechanistically, this transformation proceeds via a pathway similar to the Shi protocol, involving a sequential SET oxidation of the benzylic donor to generate benzyl radical and cation intermediates, which then engage in electrophilic substitution at the indole C3 position [32]. A key feature of this synthesis is the critical role of the *N*,*N*‐dimethylcarbamoyl (DMC) auxiliary, which not only directs the regioselectivity toward C3‐alkylation but also stabilizes the reactive intermediates, thereby enhancing overall reaction efficiency. The protocol displays broad substrate tolerance across various diphenylmethane derivatives and offers practical advantages such as operational simplicity, low cost, and the environmentally benign nature of iron catalysis.

**SCHEME 27:**
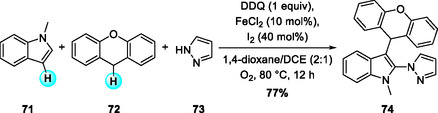
Iron‐iodine cocatalyzed oxidative intermolecular 2‐amino‐3‐alkylation of indoles with azoles and diarylalkanes.

Following the Fe/DDQ redox activation strategy, Zhang, Liang, and coworkers in 2022 developed an iron–iodine cocatalyst system promotes intermolecular aminoalkylation of indoles with azoles and diarylalkanes at the C2 and C3 positions in the presence of DDQ and molecular oxygen, affording 2‐amino‐3‐alkylindoles via a tandem C—N/C—C bond‐forming sequence (Scheme 27) [35]. The reaction exhibited broad substrate scope, showing tolerance to various functional groups, compatibility with N‐unprotected indoles, diverse xanthenes, and multiple azoles. Mechanistic studies revealed a stepwise sequence in which I_2_ first installs an azole unit at the indole C2 position, followed by DDQ/Fe/O_2_‐mediated oxidation to generate a benzylic cation. Subsequent electrophilic attack of the C2‐azolylindole on this benzylic cation furnishes the difunctionalized products.

**SCHEME 28:**
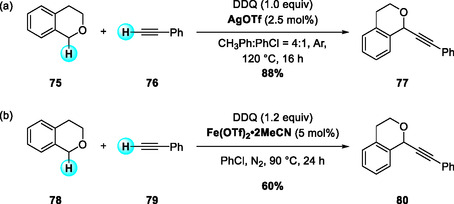
Metal‐catalyzed CDC of terminal aromatic alkynes with benzylic ethers. (a) AgOTf‐catalyzed CDC; (b) Fe(OTf)_2_‐catalyzed CDC.

In 2010, Correia and Li reported an AgOTf‐catalyzed CDC of terminal aromatic alkynes with benzylic ethers, affording alkynylmethylated ethers (Scheme 28a) [36]. The substrate scope included aryl alkynes bearing either electron‐donating or electron‐withdrawing substituents. Among benzyl ether donors, isochroman proved to be the most reactive, while methyl benzyl ether afforded low yields and dibenzyl sulfide remained unreactive. Two years later, Jiao and coworkers described a related DDQ‐assisted CDC reaction employing Fe(OTf)_2_·2MeCN instead of AgOTf as the catalyst (Scheme 28b) [37]. This modification expanded the scope to a wider range of terminal aryl alkynes and benzylic substrates. Interestingly, *o‐* and *m‐*methyl‐substituted alkynes gave higher yields than the *p*‐methyl analog, and bromo substituents were well tolerated, enabling further derivatization. Mechanistically, both reactions proceed via dehydrogenative oxidation of the benzylic ether to generate an oxocarbenium intermediate. However, the pathways differ in the mode of alkyne activation: in the AgOTf‐catalyzed system, silver acetylide is formed through direct deprotonation of the terminal alkyne. In contrast, the Fe(OTf)_2_/DDQ system relies on Fe(II)/DDQ‐mediated single‐electron oxidation/hydrogen abstraction and deprotonation of the alkyne by the reduced DDQ species, without the involvement of a metal‐acetylide intermediate.

**SCHEME 29:**
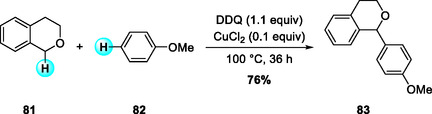
CuCl_2_‐catalyzed oxidative arylation (CDC) of isochromans.

In the same year, Todd and coworkers reported a CuCl_2_‐catalyzed oxidative arylation (CDC) of isochroman with anisole derivatives (Scheme 29) [38]. Similar to the alkynylation reactions described above, this transformation employs isochroman as a benzylic donor but utilizes aromatic compounds such as anisoles as direct coupling partners. They found that CuCl_2_ exhibited superior catalytic performance compared to other transition‐metal catalysts. The substrate scope study revealed good tolerance toward methyl‐substituted anisoles and specific naphthyl nucleophiles. This reaction represents a solvent‐free, one‐pot alternative to the traditional oxa‐Pictet–Spengler route.

**SCHEME 30:**
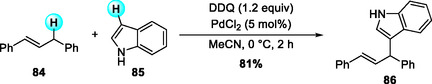
PdCl_2_‐catalyzed oxidative CDC indolation of 1,3‐diarylpropenes.

In 2009, Bao and Mo reported a PdCl_2_‐catalyzed oxidative CDC that achieves direct indolation of allylic sp^3^ C—H bonds in 1,3‐diarylpropenes with indoles (Scheme 30) [39]. The reaction proceeds efficiently at low temperature with short reaction times. It also exhibits a broad substrate scope: indoles bearing electron‐withdrawing substituents provided excellent yields, while those with electron‐donating groups required PPh_3_ as an additive to facilitate coupling, highlighting the versatility of this Pd/DDQ system.

**SCHEME 31:**
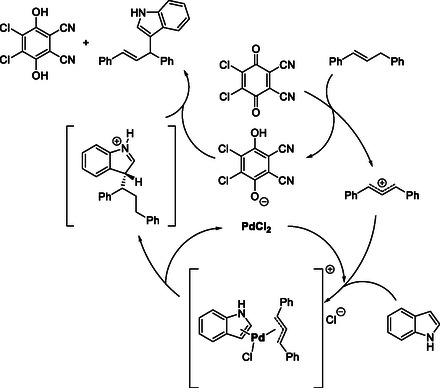
Proposed mechanism for PdCl_2_‐catalyzed oxidative CDC indolation.

Mechanistic studies suggest that DDQ abstracts a hydride from the diarylpropene to generate a conjugated cationic intermediate, which subsequently undergoes carbopalladation with PdCl_2_ and indole to form a π‐allylpalladium intermediate (Scheme 31). Subsequent elimination and proton abstraction by the reduced DDQ (phenolate anion) afford the C—C coupled indole product. The oxidation state of palladium remains unchanged throughout the catalytic cycle, as DDQ serves as the sole redox mediator responsible for substrate oxidation.

**SCHEME 32:**
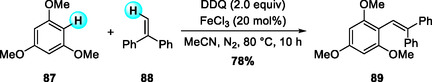
DDQ‐mediated, FeCl_3_‐catalyzed oxidative C—H/C—H cross‐coupling between electron‐rich arenes and alkenes.

Expanding upon the metal catalyst/DDQ‐mediated allylic coupling chemistry, Lei and coworkers (2015) demonstrated a DDQ/FeCl_3_‐catalyzed oxidative arene–alkene cross‐coupling that affords triarylethylenes in high yields (Scheme 32) [40]. FeCl_3_ likely enhances DDQ's oxidative strength or activates the alkene toward electrophilic substitution, enabling efficient metal‐assisted C—H/C—H bond coupling.

**SCHEME 33:**
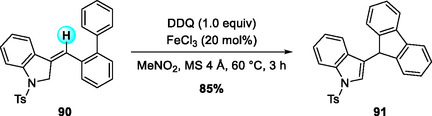
DDQ/FeCl_3_‐mediated tandem oxidation of biaryl‐tethered 3‐(methylene)indolines.

Building upon previous Fe/DDQ oxidative coupling strategies, Jana and coworkers (2021) introduced a tandem oxidation that generates indole–fluorene hybrids from biaryl‐tethered 3‐(methylene)indolines with high regioselectivity and excellent yields (Scheme 33) [9]. The reaction sequence involves allylic sp^3^ C—H oxidation followed by intramolecular C—C bond formation, efficiently furnishing complex indole–fluorene architectures under mild conditions. Although the precise role of FeCl_3_ remains unclear, experimental evidence suggests that it increases the availability of the indolyl cation intermediate by promoting reversible dehydration and suppressing undesired byproduct formation.

**SCHEME 34:**
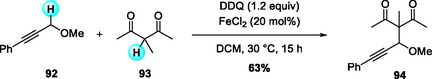
DDQ‐mediated, iron‐catalyzed alkylation of propargyl ethers with 1,3‐dicarbonyl derivatives.

Next, the metal catalyst can act as a Lewis acid to activate nucleophiles in DDQ‐mediated transformations. In 2011, Zhang and coworkers reported an iron‐catalyzed alkylation of the sp^3^ C—H bond α to oxygen in propargyl ethers with 1,3‐dicarbonyl compounds, affording β‐dicarbonyl ethers (Scheme 34) [41]. The substrate scope includes aryl propargyl methyl ethers bearing both electron‐donating and electron‐withdrawing substituents, as well as propargyl allyl and benzyl ethers. The substate scope extends to both linear and cyclic 1,3‐dicarbonyl compounds, with the latter giving diastereomeric mixtures. In this system, iron salts primarily function as Lewis acids that activate 1,3‐dicarbonyl compounds toward nucleophilic addition.

**SCHEME 35:**
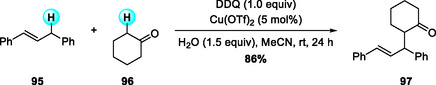
Cu(OTf)_2_‐catalyzed DCC between 1,3‐diarylpropenes and ketones.

In 2014, Huang and coworkers developed a Cu(OTf)_2_‐catalyzed dehydrogenative cross‐coupling (DCC) between allylic C—H bonds and the α‐C—H bonds of ketones or aldehydes to synthesize γ,δ‐unsaturated carbonyl compounds (Scheme 35) [42]. The reaction proceeds under mild conditions and exhibits broad substrate tolerance, accommodating a variety of simple ketones and aldehydes. In this transformation, the copper catalyst functions as a Lewis acid to activate the carbonyl compound, forming a copper–enolate intermediate that subsequently couples with the allylic component. This strategy overcomes the limitations of previous methods that were largely restricted to electron‐rich arenes or alkynes, providing a practical and efficient route for C—C bond formation.

**SCHEME 36:**
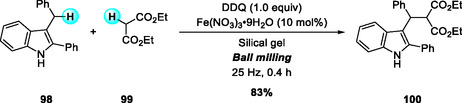
Fe(NO_3_)_3_·9H_2_O‐catalyzed solvent‐free CDC of 3‐benzylic indoles with acidic methylenes.

In 2016, Su and coworkers reported a DDQ‐mediated, Fe(NO_3_)_3_·9H_2_O‐catalyzed, solvent‐free CDC of 3‐benzylic indoles with acidic methylenes to afford 3‐arylmethylindoles and bisindole derivatives under high‐speed ball‐milling at room temperature (Scheme 36) [43]. The protocol employs inexpensive and environmentally benign Fe(III) salts with DDQ or TBHP as oxidants, achieving efficient C—C bond formation between N‐heterocycles and active methylene substrates. This work demonstrates the potential of mechanochemical activation as a simple and sustainable route for oxidative CDC reactions.

**SCHEME 37:**
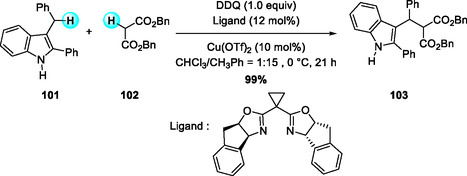
Cu(OTf)_2_/bis(oxazoline)‐catalyzed enantioselective oxidative cross‐coupling of 3‐indolylmethyl sp^3^ C—H bonds with 1,3‐dicarbonyl derivatives.

Following the development of catalyst/DDQ‐mediated oxidative coupling systems stereoselective C—C bond formations have been extensively investigated. In 2010, Gong and coworkers reported the first Cu(OTf)_2_/bis(oxazoline)‐catalyzed enantioselective oxidative cross‐coupling of 3‐indolylmethyl sp^3^ C—H bonds with 1,3‐dicarbonyl compounds (Scheme 37) [44]. The reaction exhibits broad substrate scope, tolerating diverse indoles and 1,3‐dicarbonyl derivatives, and provides a practical, atom‐efficient route to enantioselective C—C bond formation. The chiral Cu–bis(oxazoline) complex functions as a Lewis acid, enhancing the oxidizing ability of DDQ and controlling the stereochemical course of nucleophilic addition in the transition state.

**SCHEME 38:**
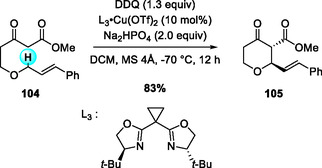
DDQ‐mediated, Cu(OTf)_2_/bis(oxazoline)‐catalyzed intramolecular CDC of β‐ketoesters with cinnamyl/benzyl ethers.

In 2018, Scheidt and coworkers reported a Cu(OTf)_2_/bis(oxazoline)‐catalyzed intramolecular CDC of β‐ketoesters bearing cinnamyl or benzyl ethers, affording enantioenriched tetrahydropyran‐4‐ones (Scheme 38) [45]. Under diluted conditions, the Cu(II)–bis(oxazoline) complex (ligand L3) furnished the desired disubstituted tetrahydropyranones in high yields and with excellent enantiomeric ratios (up to 97:3). The reaction displays a broad substrate scope, tolerating substrates bearing electron‐donating or electron‐withdrawing substituents on the aromatic rings, as well as naphthyl and trisubstituted cinnamyl derivatives. Furthermore, subsequent transformations of the β‐ketoester products provide efficient routes to tetrahydrofuran scaffolds frequently featured in biologically active molecules.

**SCHEME 39:**
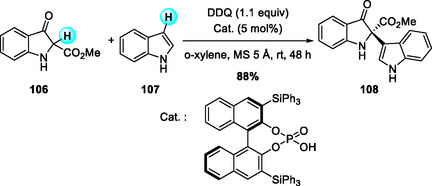
Organocatalytic enantioselective aza‐Friedel–Crafts alkylation of indoles with indolenines.

In 2018, Basireddy and coworkers reported a DDQ‐promoted enantioselective aza‐Friedel–Crafts alkylation of indoles with indolenines generated in situ from 3‐indolinone‐2‐carboxylates, catalyzed by a chiral BINOL‐derived phosphoric acid (Scheme 39) [46]. This organocatalytic approach operates under mild conditions and efficiently furnishes chiral indol‐3‐yl‐3‐indolinone‐2‐carboxylate derivatives in good yields and with excellent enantioselectivities (up to 98.6% ee). The reaction features broad substrate compatibility, including various indole and indolinone electrophiles; pyrrole and 3‐substituted indoles also serve as viable nucleophiles, although electron‐rich substrates show somewhat reduced reactivity. Application to the assembly of the trigonoliimine C core highlights its utility for accessing indolinone alkaloids.

**SCHEME 40:**
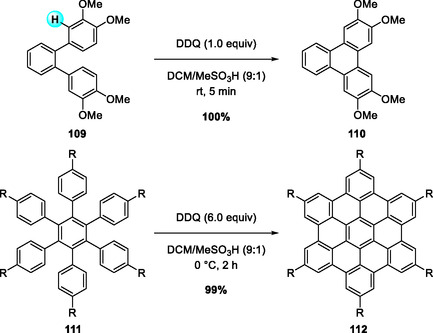
DDQ‐mediated (DDQ/H^+^) Scholl oxidation of polyaromatic precursors.

DDQ‐promoted C—C bond formation under Brønsted acid conditions has also been explored through radical‐cation pathways. In 2009, Rathore and coworkers reported a DDQ/H^+^‐mediated Scholl oxidation that enables oxidative C(sp^2^)—C(sp^2^) bond formation—particularly biaryl coupling—both intra‐ and intermolecularly from polyaromatic precursors (Scheme 40) [8]. The DDQ/H^+^ combination enhances the oxidation potential of DDQ, facilitating the generation of radical‐cation intermediates that couple at aromatic C—H sites. This method was effectively applied to the synthesis of extended polycyclic aromatics such as hexa‐peri‐hexabenzocoronene and addressed common issues in classical Scholl reactions requiring harsh chlorinating oxidants like FeCl_3_, MoCl_5_, or SbCl_5_.

**SCHEME 41:**
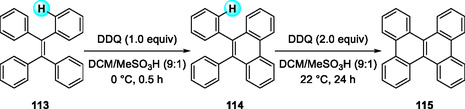
DDQ/H^+^‐mediated sequential oxidative transformation of symmetrical/unsymmetrical tetraarylethylenes.

Two years later, the same group reported another DDQ/H^+^‐mediated sequential oxidative transformation of symmetrical and unsymmetrical tetraarylethylenes (TAPs) into 9,10‐diarylphenanthrenes (DAPs) and dibenzochrysenes (DBCs) [47]. The DDQ/H^+^ system effectively oxidizes a wide range of aromatic electron donors—exhibiting oxidation potentials up to ≈1.6 V versus SCE—into their corresponding radical cations [8, 48]. The reaction proceeds under mild conditions in CH_2_Cl_2_/MeSO_3_H (9:1) with 1 or 2 equivalents of DDQ (Scheme 41). The selective formation of DAPs with 1 equivalent of DDQ, without any dibenzochrysene contamination, strongly supports a stepwise oxidation pathway involving paramagnetic radical cations as key intermediates.

**SCHEME 42:**
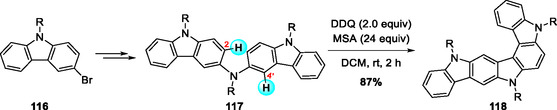
DDQ/MSA‐mediated intramolecular oxidative cyclization of carbazole dimer precursors.

In 2022, Iwanaga and coworkers reported the synthesis of π‐extended carbazole dimers via an intramolecular oxidative cyclization of nitrogen‐linked bis(carbazole) frameworks under DDQ/sulfonic acid conditions (Scheme 42) [49]. Spectroscopic and electrochemical studies ruled out a radical‐cation mechanism, instead supporting a dicationic pathway involving two‐electron oxidation. Mulliken charge analysis based on DFT calculations revealed a positive charge at the 2‐position and a negative charge at the 4′‐position in the dicationic species, rationalizing the observed preference for 2,4′‐bond formation [50].

In summary, catalyst‐assisted DDQ‐mediated C—C bond formation typically involves transition metals (Fe, Cu, Ag, Pd, etc.) serving as the primary oxidation or activation centers, while DDQ functions as a co‐oxidant and electron acceptor to generate cation intermediates such as oxocarbenium or iminium ions that drive bond construction. This approach encompasses a broad substrate range, including benzylic, allylic, and olefinic C—H bonds, and enables diverse transformations such as arylation, alkynylation, and alkylation, depending on the metal–substrate combination. In some cases, the metal catalyst acts as a Lewis acid to activate nucleophiles. Within acid‐promoted Scholl or cyclization systems, both single‐electron and two‐electron (dicationic) pathways have been demonstrated. Collectively, these studies establish the DDQ/metal catalytic ensemble as a versatile and practical methodology for CDC, oxidative substitution or cyclization, and other selective C—C bond‐forming transformations.

## 3 DDQ‐Catalyzed C—C Bond Formation

### 3.1 Thermal DDQ‐Catalyzed C—C Bond Formation

DDQ itself has also been employed as an efficient catalyst for C—C bond‐forming reactions. These processes typically rely on electron‐transfer pathways, and can proceed under thermal, photochemical, or electrochemical conditions. In general, a catalytic amount of DDQ (or its activated form) oxidizes the substrate to generate radical or radical‐cation intermediates that subsequently undergo bond formation. The reduced DDQ is then reoxidized by an external oxidant, completing the catalytic cycle.

**SCHEME 43:**
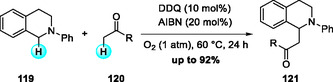
DDQ‐catalyzed oxidative CDC of N‐aryl THIQs with ketone derivatives.

In 2012, Prabhu and coworkers reported a DDQ‐catalyzed oxidative CDC of N‐aryl THIQs with ketone derivatives under O_2_ in the presence of AIBN (Scheme 43) [51]. A wide range of N‐aryl THIQs efficiently coupled with simple ketones under mild, metal‐free conditions, affording β‐amino carbonyl products within short reaction times.

**SCHEME 44:**
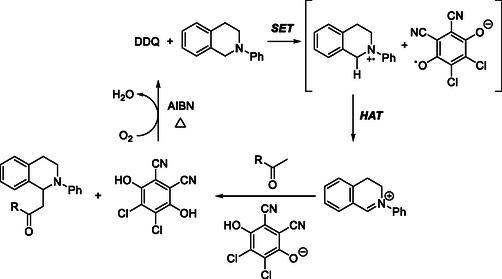
Proposed mechanism of DDQ‐catalyzed oxidative CDC of N‐aryl THIQs.

Mechanistically, DDQ oxidizes N‐aryl THIQs through a SET and HAT sequence, generating an iminium ion along with the reduced DDQ anion. The latter deprotonates the α‐position of the ketone to produce the corresponding enolate nucleophile, which subsequently engages in a Mannich‐type C—C bond formation with the iminium intermediate to afford the β‐amino carbonyl adduct. Finally, the reduced DDQH_2_ is reoxidized by AIBN/O_2_, thereby regenerating the catalytic cycle (Scheme 44).

**SCHEME 45:**
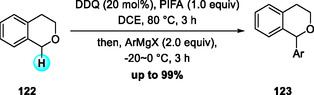
DDQ‐catalyzed sp^3^ C—H functionalized arylation of isochromans.

Following their earlier stoichiometric DDQ‐mediated C—C bond‐forming reactions of isochromans with Grignard reagents (Scheme 4a) [12, 13], Muramatsu and coworkers later developed a catalytic version that achieves sp^3^ C—H functionalization of isochromans under DDQ catalysis (Scheme 45) [52]. Optimization studies identified PIFA as the most effective terminal oxidant, with the reaction proceeding efficiently in DCE at 80°C followed by addition of Grignard reagent at low tempearture. This method provides arylation with aryl Grignard reagents and further extends to alkylation, allylation, amidation (TsNH_2_, TsNHMe), and azidation (NaN_3_), thereby demonstrating broad nucleophile compatibility and significantly expanded synthetic scope.

**SCHEME 46:**
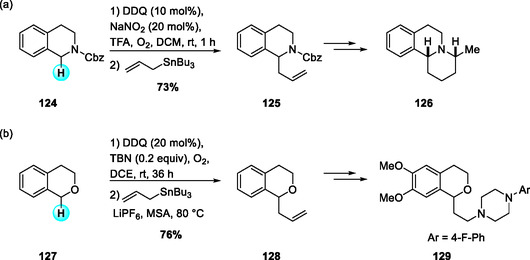
Aerobic DDQ‐catalyzed allylation reaction of N‐Cbz THIQs (a) and isochromanes (b) with allylstannane.

In 2022, Min and coworkers reported an aerobic DDQ‐catalyzed allylation of N‐Cbz THIQs with allylstannanes under TFA and NaNO_2_ in the presence of molecular oxygen (Scheme 46a) [53, 54]. While the stoichiometric DDQ‐mediated allylation of THIQs had been disclosed earlier in 2020 [54], this catalytic protocol efficiently promoted C(sp^3^)—H functionalization with both electron‐rich and electron‐deficient substrates. The allylated products were transformed into benzo[a]quinolizidines as single diastereomers through sequential metathesis and hydrogenation. Two years later, the same group developed a related DDQ‐catalyzed aerobic α‐allylation of isochromanes with allylstannanes, employing tert‐butyl nitrite (TBN) as an oxidation mediator for DDQ regeneration (Scheme 46b) [55]. This method proceeds through reversible oxocarbenium ion formation followed by efficient allylstannane trapping, and was further applied to the synthesis of natural product‐like relevant heterocyclic scaffolds [56].

**SCHEME 47:**
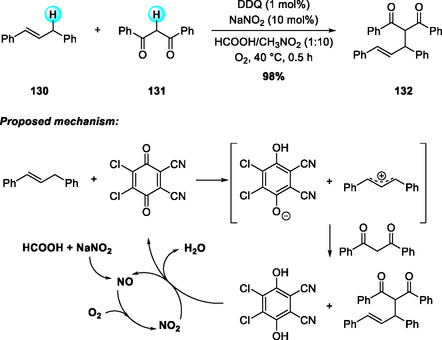
DDQ/NaNO_2_‐catalyzed oxidative coupling of diarylpropenes with 1,3‐dicarbonyls.

In 2015, Yan and coworkers reported a highly efficient DDQ/NaNO_2_‐catalyzed oxidative coupling of diarylpropenes with 1,3‐dicarbonyl compounds under molecular oxygen (Scheme 47) [57]. Notably, this transformation proceeds with only 1 mol% of DDQ—significantly lower than the typical 10 mol% required in similar catalytic systems. The reaction merges benzylic or allylic C(sp^3^)—H donors with active methylene nucleophiles under metal‐free and aerobic conditions, affording C—C coupled products in good yields. Mechanistically, DDQ oxidizes the benzylic C—H bond via hydride abstraction or a SET/HAT sequence to generate an allylic cation, which is trapped by the 1,3‐dicarbonyl enolate. In parallel, NaNO_2_ and formic acid produce NO, which is oxidized to NO_2_ by oxygen; the resulting NO_2_ reoxidizes DDQH_2_ to DDQ, sustaining the catalytic cycle while minimizing oxidant consumption.

**SCHEME 48:**
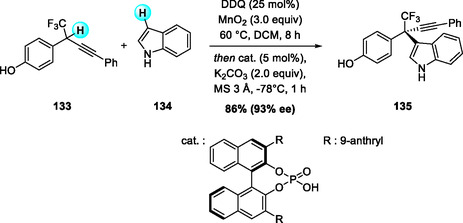
Organocatalytic enantioselective CDC of *p*‐hydroxybenzyl‐CF_3_ substrates with indoles.

In 2020, Liu and coworkers reported an enantioselective CDC of racemic *p*‐hydroxybenzyl‐CF_3_ substrates with indoles using a DDQ/MnO_2_ cocatalytic system and a chiral phosphoric acid (Scheme 48) [58]. Oxidation of the substrate with stoichiometric DDQ alone was found to be reversible, while introduction of MnO_2_ as a terminal oxidant provides continuous regeneration of DDQ from DDQH_2_, effectively driving the catalytic cycle. The chiral phosphate promotes 1,6‐addition of indoles to the *p*‐quinone methide (*p*‐QM) intermediate, affording CF_3_‐substituted all‐carbon quaternary centers with high enantioselectivity. The method also extends to poly‐ and perfluoroalkyl analogs (CF_2_Cl, C_2_F_5_, C_3_F_7_), highlighting its utility for asymmetric construction of fluorinated quaternary stereocenters.

**SCHEME 49:**
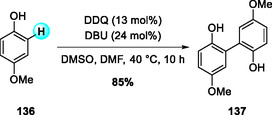
DDQ/DBU‐catalyzed radical protocol for efficient synthesis of lignin‐derived biphenyl (biaryl) dimers.

In 2025, Du and coworkers reported a DDQ/DBU‐catalyzed radical protocol for sp^2^ C—H activation, enabling efficient synthesis of lignin‐derived biaryl dimers under mild conditions (Scheme 49) [59]. Model studies with guaiacol confirmed selective S–S (eugenol) and G–G (guaiacol) couplings, as verified by 2D NMR analysis. The reaction proceeded with comparable efficiency under Ar, air, or O_2_, while DMSO served a dual role as both a yield enhancer and a selectivity modulator. The optimized system achieved 75%–85% conversions for various *p*‐methoxyphenol derivatives and was further extended to native lignin feedstocks, offering a scalable and sustainable route to biaryl synthesis. This metal‐free strategy provides a green and practical approach to lignin valorization and the generation of functional aromatic frameworks.

In short, thermal DDQ catalysis enables efficient C—C bond formation between benzyl or allyl C(sp^3^)—H and aromatic C(sp^2^)—H sites through either oxocarbenium/iminium‐mediated nucleophilic capture or radical pathways. In the THIQ and isochroman series, DDQH_2_ is reoxidized to DDQ by O_2_/radical initiators or NO sources (TFA/NaNO_2_, TBN), accommodating a wide range of carbon nucleophiles such as allylstannanes, Grignard reagents, and 1,3‐dicarbonyl compounds. Moreover, MnO_2_‐assisted enantioselective CDC and DBU‐promoted aromatic dimerization further exemplify the versatility of this DDQ‐driven catalytic strategy. Collectively, these advances demonstrate high efficiency, broad functional‐group tolerance, and late‐stage applicability under mild and sustainable conditions.

### 3.2 Photoinduced DDQ‐Catalyzed C—C Bond Forming Reactions

**SCHEME 50:**
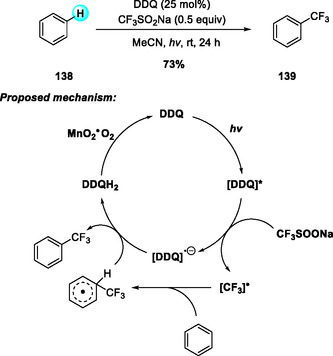
Visible‐light‐induced DDQ‐catalyzed trifluoromethylation of arenes and heteroarenes with CF_3_SO_2_Na.

Visible‐light‐induced trifluoromethylation of aromatic compounds using CF_3_SO_2_Na was first demonstrated in 2013 with anthraquinone‐2‐carboxylic acid as the photocatalyst [60]. Inspired by this precedent, Yuan and coworkers (2017) developed a DDQ‐catalyzed photoredox system that enables efficient trifluoromethylation of arenes and heteroarenes under visible light (Scheme 50) [61]. The reaction proceeds smoothly in air at room temperature, tolerating both electron‐rich and electron‐deficient substrates. Mechanistic studies reveal that photoexcited DDQ generates CF_3_ radicals, which add to the arene followed by rearomatization through DDQ–semiquinone–hydroquinone cycling. Regeneration of DDQ from its reduced form by fixed‐bed MnO_2_ provides a practical and sustainable oxidation system.

**SCHEME 51:**
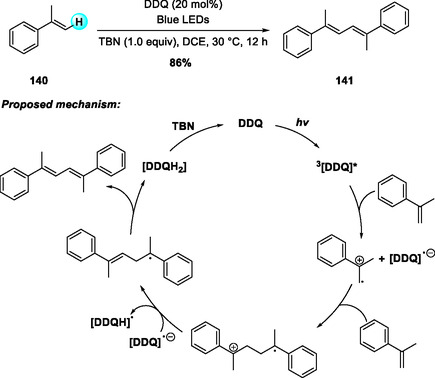
Visible‐light DDQ‐photocatalyzed dehydrogenative homodimerization of mono‐ and disubstituted olefins.

In 2018, Natarajan and coworkers reported a visible‐light DDQ‐photocatalyzed dehydrogenative homodimerization of mono‐ and disubstituted olefins to form symmetrical buta‐1,3‐dienes (Scheme 51) [62]. The reaction proceeds efficiently under blue LED irradiation in DCE, with superior performance under N_2_ compared to air or O_2_, where radical quenching leads to peroxidic byproducts [63, 64]. A wide substrate scope was observed, favoring electron‐rich olefins over electron‐deficient analogs. Mechanistically, photoexcited triplet DDQ acts as a potent oxidant, generating benzylic radical cations that couple to form the dimeric adduct. Subsequent proton abstraction and HAT furnish the conjugated diene, while DDQH_2_ is reoxidized to DDQ by TBN, maintaining the catalytic cycle.

**SCHEME 52:**
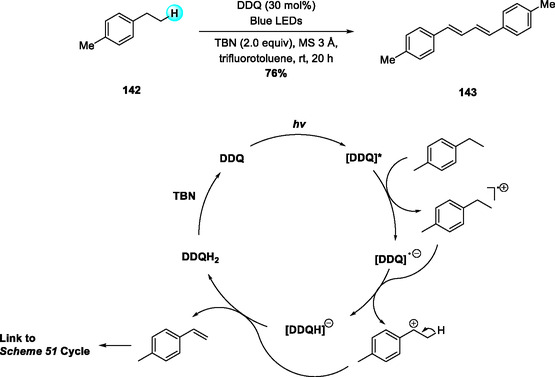
Visible‐light DDQ‐catalyzed olefination–dimerization of alkylbenzenes.

In 2022, Chuskit and coworkers reported a visible‐light DDQ‐photocatalyzed one‐pot synthesis of symmetrical buta‐1,3‐dienes via tandem oxidative dehydrogenation and dimerization of alkylbenzenes (Scheme 52) [65]. The reaction proceeds efficiently under blue LED irradiation at room temperature, with DDQ serving as the photooxidant for both steps and TBN enabling continuous DDQ regeneration. Under optimized conditions in trifluorotoluene under blue light, the protocol exhibits broad substrate scope and delivers α,α‐diarylmethane‐derived dienes in excellent yields even on gram scale.

Under visible‐light irradiation, DDQ acts as a potent photocatalyst that facilitates controlled radical generation through its high excited‐state oxidation potential and reversible semiquinone/hydroquinone cycling. Trifluoromethylation of arenes and heteroarenes with CF_3_SO_2_Na proceeds efficiently across both electron‐rich and electron‐deficient substrates, with reoxidation of DDQ‐mediated by MnO_2_. DDQ photocatalysis also promotes dehydrogenative coupling of olefins and tandem oxidative dimerization of alkylbenzenes under blue LEDs, with TBN assisting in DDQ regeneration. Collectively, these transformations highlight DDQ's superior photooxidizing capability and clean redox cycling, providing an effective route to selective C—C bond formation and sustainable synthesis under mild, operationally simple conditions.

### 3.3 Electrochemical DDQ‐Catalyzed C—C Bond Formation

**SCHEME 53:**
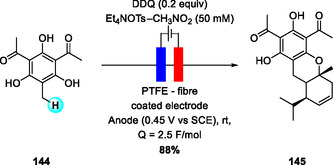
Electrochemical, DDQH_2_‐mediated hetero‐Diels–Alder cycloaddition of grandinol and terpenes.

Only a few examples of C—C bond‐forming reactions employing DDQ as an electrochemical mediator have been reported. In 1998, Tada and coworkers described an electrochemical DDQ‐catalyzed hetero‐Diels–Alder cycloaddition between grandinol and various terpenes, affording multiple euglobal derivatives under mild conditions (Scheme 53) [66]. The transformation, conducted on a PTFE‐fiber‐coated electrode, allows precise oxidative activation of phenolic substrates with minimal oxidant load, achieving high selectivity and reusability of the electrode.

**SCHEME 54:**
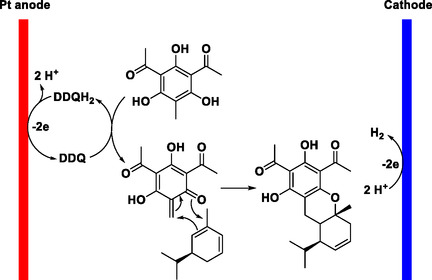
Proposed mechanism for electrochemical, DDQH_2_‐mediated hetero‐Diels–Alder cycloaddition.

Under constant‐potential electrolysis, DDQH_2_ is anodically oxidized to DDQ, while protons are reduced to hydrogen at the platinum cathode (Scheme 54). The oxidized DDQ species then converts the phenolic substrate into a reactive *O*‐quinone intermediate, which engages in a hetero‐Diels–Alder reaction with the terpene to form the euglobal core. The mediator is continuously regenerated at the anode, maintaining the DDQ redox cycle. This study established an early biomimetic model for electrochemically driven DDQ‐catalyzed cycloadditions.

**SCHEME 55:**
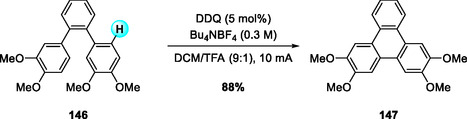
Electrochemical, DDQ‐catalyzed intramolecular Scholl oxidative coupling of polyaromatic precursors to polyphenylenes.

In 2017, Hilt and coworkers reported an electrochemical DDQ‐catalyzed intramolecular Scholl oxidative coupling of polyaromatic precursors to polyphenylenes (Scheme 55) [67]. The methodology accommodates terphenyls, quaterphenyls, and higher oligophenylenes up to coronene frameworks, affording planar polycyclic aromatics efficiently. Cyclic voltammetry confirmed DDQ's role as a redox mediator, while direct electrolysis in the absence of DDQ resulted in diminished reactivity. This electrochemical approach provides a scalable and sustainable alternative for Scholl‐type oxidative construction of extended polycyclic aromatic hydrocarbons (PAHs) without prefunctionalization.

**SCHEME 56:**
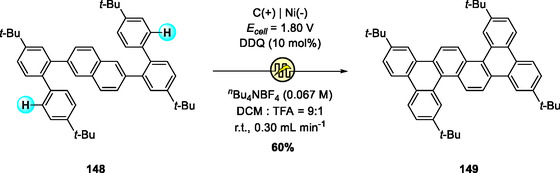
DDQ‐catalyzed e‐flow Scholl reaction for synthesis of PAHs.

Expanding on the electrochemical Scholl methodology, Li and Ye recently developed an electrochemical flow (e‐flow) Scholl reaction for the synthesis of twisted benzo‐extended [n]phenacenes, exemplified by **149** (Scheme 56) [68]. The reaction, employing DDQ as a catalytic redox mediator under mild anodic conditions, efficiently produced **149** in significantly higher yield compared to conventional Scholl oxidations using stoichiometric FeCl_3_ or DDQ/TFA. Notably, the continuous‐flow setup allowed straightforward scale‐up by simply extending electrolysis time without altering reaction parameters, highlighting the advantages of electrochemical DDQ catalysis for efficient and sustainable PAH synthesis [69].

Electrochemical DDQ catalysis enables C—C bond formation via anodic DDQ/DDQH_2_ redox cycling under mild and scalable conditions. From Tada's mediated cycloadditions to Hilt's and Li–Ye's electrochemical and flow‐based Scholl oxidations, these methods demonstrate sustainable and controllable access to polyaromatic frameworks.

### 3.4 Miscellaneous DDQ‐Promoted C—C Bond Formation

**SCHEME 57:**
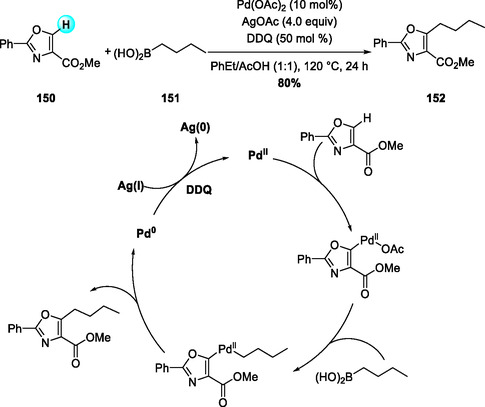
Pd(II)‐catalyzed direct C5‐alkylation of oxazoles with alkylboronic acids using DDQ as an oxidant.

In certain catalytic systems, DDQ serves primarily as a redox turnover promoter rather than a direct oxidant. Li and coworkers (2017) reported a DDQ‐assisted Pd(II)‐catalyzed C5‐alkylation of oxazoles with alkylboronic acids via C(sp^2^)—H/C(sp^3^)—B activation (Scheme 57) [70]. DDQ efficiently reoxidizes Pd(0) to Pd(II) in cooperation with Ag(I), surpassing benzoquinone in catalytic turnover efficiency. The reaction exhibits broad functional‐group tolerance across 2‐phenyloxazole‐4‐carboxylates and accommodates both primary and secondary alkylboronic acids. This work represents the unusual example of C(sp^2^)—C(sp^3^) coupling at the oxazole C5‐position, highlighting its potential for late‐stage diversification of oxazole scaffolds.

**SCHEME 58:**
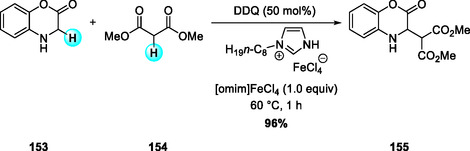
DDQ‐catalyzed C(sp^3^)—C(sp^3^) coupling in [omim]FeCl_4_ ionic liquid.

In 2022, Mirzaei and coworkers reported a DDQ‐catalyzed C(sp^3^)—C(sp^3^) CDC of 1,4‐benzoxazol‐2‐one with malonic acid esters conducted in the ionic liquid [omim]FeCl_4_ (Scheme 58) [71]. The medium functions simultaneously as solvent and catalyst, providing high reactivity, recoverability, and reusability. The reaction tolerates diverse N‐benzyl benzoxazin‐2‐one and malonate derivatives, maintaining consistent yields across substituent types. Replacement of the ionic liquid with MeCN/FeCl_3_ led to diminished efficiency, underscoring the synergistic role of the ionic liquid in promoting DDQ catalysis.

## 4 Summary and Outlook

This review has summarized the diverse roles of DDQ as an efficient and versatile oxidant for C—C bond formation, organized according to stoichiometric (including co‐oxidant) and catalytic modes encompassing thermal, photochemical, and electrochemical systems. Under stoichiometric conditions, DDQ serves as a powerful hydride or electron acceptor, enabling a variety of oxidative transformations such as CDC, dehydrogenation, oxidative cyclization, and the construction of extended polycyclic aromatics. In catalytic systems, DDQ operates through distinct redox cycles—thermal regeneration, photoinduced SET, or electrochemical oxidation—allowing controlled activation of C(sp^2^)— or C(sp^3^)—H bonds under mild conditions.

From a practical standpoint, DDQ offers a valuable alternative to metal‐based oxidants, minimizing the use of transition metals and associated environmental concerns. Its tunable redox potential, good solubility, and acid stability provide high reproducibility and selectivity in oxidative coupling processes. Reactions typically proceed at ambient temperature with minimal byproducts, highlighting DDQ's utility in sustainable synthesis.

Despite these advances, the scope of DDQ‐catalyzed C—C bond construction remains relatively narrow compared to the reported studies on C—N and C—O bond formation. Most existing examples focus on aromatic systems, whereas aliphatic and asymmetric transformations are still underdeveloped. Future opportunities lie in expanding DDQ catalysis toward asymmetric C—C coupling, particularly by integrating chiral Brønsted acids or organocatalysts, and in exploiting photo‐ and electrochemical pathways for more energy‐efficient oxidation. The design of new DDQ derivatives with fine‐tuned redox potentials, guided by computational studies, may further enhance selectivity and substrate compatibility.

Looking ahead, the combination of experimental and theoretical advances is expected to transform DDQ‐based synthetic methodologies into a selective, broadly applicable, and sustainable approach to controlled oxidative C—C bond construction, with broad potential in both laboratory and industrial applications.

## Funding

This work was supported by National Research Foundation of Korea (RS‐2025‐16069848 and RS‐2025‐02213941).

## Conflicts of Interest

The authors declare no conflicts of interest.
